# No Solid Colloidal Carriers: Aspects Thermodynamic the Immobilization Chitinase and Laminarinase in Liposome

**DOI:** 10.3389/fbioe.2021.793340

**Published:** 2022-02-07

**Authors:** Dania Alonso-Estrada, Nayra Ochoa-Viñals, Sandra Pacios-Michelena, Rodolfo Ramos-González, Arianna Núñez-Caraballo, Lourdes Georgina Michelena Álvarez, José Luis Martínez-Hernández, Alberto Antonio Neira-Vielma, Anna Ilyina

**Affiliations:** ^1^ Nanobioscience and Biological and Genomic Sciences Research Groups, Postgraduate Program in Food Science and Technology, Faculty of Chemical Sciences of the Autonomous University of Coahuila, Saltillo, México; ^2^ CONACYT- Autonomous University of Coahuila, Postgraduate Program in Food Science and Technology, Faculty of Chemical Sciences of the Autonomous University of Coahuila, Saltillo, México; ^3^ Faculty of Industrial Engineering, Autonomous University of Durango, Durango, México; ^4^ Cuban Institute for Research on Sugarcane Derivatives (ICIDCA), Havana, Cuba

**Keywords:** liposomes, micelles, nanoemulsions, bilosomes, ethosomes, niosomes, layersomes, transfersomes

## Abstract

The present review describes the basic properties of colloidal and vesicular vehicles that can be used for immobilization of enzymes. The thermodynamic aspects of the immobilization of enzymes (laminarinase and chitinase) in liposomes are discussed. These systems protect enzymes against environmental stress and allow for a controlled and targeted release. The diversity of colloidal and vesicular carriers allows the use of enzymes for different purposes, such as mycolytic enzymes used to control phytopathogenic fungi.

## Introduction

Enzyme encapsulation has proven its effectiveness in various applications in the biotechnology, pharmaceutical and food industries, and nanoencapsulation of enzymes is one of the most exciting areas of research now underway (Koshani and Jafari, 2019; Taheri and Jafari, 2019). Enzymes, as natural biocatalysts, serve a variety of purposes. Their usage in the industrial production of biofuels, paper, food, agriculture, medicines, fine chemicals, and feed has increased in recent decades. The majority of companies are looking for a way to increase the potential of enzymes in biotechnology applications. One of the most significant disadvantages is the enzymes’ limited stability, which may often be overcome via immobilization techniques. According to Business Communication Company Research (BCC), the worldwide industrial enzyme market is predicted to expand from $5.5 billion in 2018 to $7.0 billion in 2023. This is in line with the industry’s growing trend toward more sustainable and cost-effective methods. Because enzymes accelerate chemical processes in a very sustainable and efficient manner, their application in chemistry underlies our society’s move to a more ecological economy (Mohammadi et al., 2021).

**FIGURE 1:**
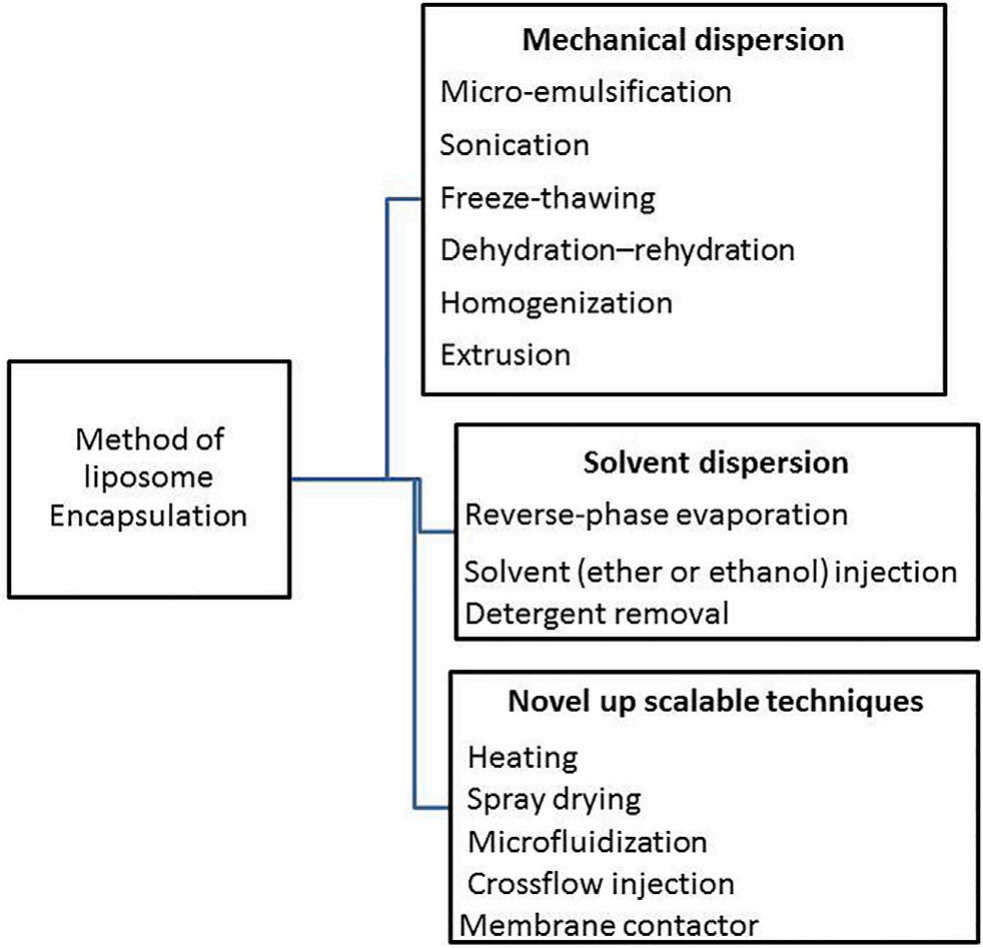
Scheme en listed the main methods applied for encapsulation on colloidal/vesicular systems.

Unfortunately, enzymes’ biological origins make it difficult for them to be used as industrial catalysts. As a result, scientists have been driven to create new technologies to optimize enzymes for commercial applications. Furthermore, enzymatic immobilization alters the enzyme activity profile as a function of temperature and pH, allowing the catalyst to be reused in many reaction cycles (Dos Santos and Dias-Souza 2016). Microencapsulation is a type of physical immobilization that doesn’t rely on the creation of covalent bonds. There are a variety of encapsulation (Figure 1) methods available that do not use solid supports but do include a colloidal phase (Jafari, 2017).

The most common hydration approach is thin-film hydration, which is ideal for encapsulating both hydrophilic and hydrophobic compounds. Multilamellar vesicle is produced using these approaches the lipid mixture is dissolved in solvents under continuous stirring and then injected into the aqueous phase under pressure in injection techniques. Liposome production without organic solvents using a microfluidizer provides very stable liposomes without quick aggregation or fusion (Maja et al., 2020). After a fine hydration, sonication is used to create tiny, small unilamellar vesicle to reduce the size of the vesicles, probe and bath sonication are commonly utilized. Sonication, on the other hand, can change the active conformation of enzymes. To obtain more homogenous and stable liposomes, a high-pressure extrusion process is used. The physical modification of the lamellar structure caused by freezing the lipid formulation gives it a final ionic configuration. The lipid phase and solvents that are chosen are crucial. The polar component of the colloid (lipid, detergents, etc.) links with a polar environment of an aqueous medium, while the hydrophobic phase is arranged in layers, as a lipid bilayer, to produce vesicles. An emulsion (water-in-oil) is formed by magnetic stirring or sonication of an instance containing phospholipids in an organic solvent such as diethylether, isopropyl ether, or combinations of these ethers with chloroform or methanol and aqueous buffer in the reverse-phase evaporation process. The organic solvent is next evaporated under decreased pressure, converting the system to aqueous liposome dispersion. The dehydration–rehydration technique is a reasonably simple liposome synthesis test. This configuration demands a significant quantity of energy (obtained from sonication, homogenization, heating, etc.). Finally, in order to select the most appropriate liposomal production method, the following parameters must be considered: 1) the physicochemical properties of the liposomal ingredients and the components that must be entrapped within liposomes; 2) the nature of the medium; 3) the most efficient concentration of the entrapped material; 4) additional procedures required during the delivery or application of the liposomes; 5) the optimum size of liposomal vesicles; and 6) the optimum required shelf-life (Rafiie and Jafari 2018).

**FIGURE 2:**
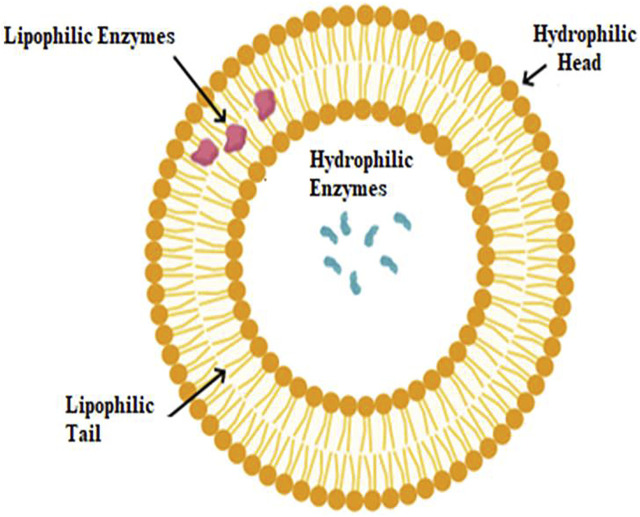
Schematic description of hydrophilic and lipophilic enzyme localization on liposomes.

Solid support free encapsulates can be classified as colloidal or vesicular vehicles based on their structural characteristics. Vesicles, double-layered amphiphilic structures with a water core, can carry hydrophilic or hydrophobic enzymes. Hydrophobic enzymes are located in the aqueous core, while hydrophilic enzymes are located within a double layer totally or partially (Figure 2).

This research looks at the properties of colloidal and vesicular carriers, as well as how they may be used to immobilize enzymes. Thermodynamic properties of chitinase and laminarinase encapsulation in soya lecithin liposomes. For spectrophotometric studies of enzyme activity, laminarin and *p*-nitrophenyl—D-N-acetyl-glucosamide were utilized as substrates.

## Properties of Colloidal and Vesicular Vehicles

The major characteristics of the solid support free systems used for encapsulation are summarized in Table 1. All of the methods mentioned above increase the surface area available for enzyme-biological substrate interactions, limit undesired interactions with proteolytic enzymes, improve solubility and absorption, protect against environmental stress, and allow for targeted and controlled release. Because of the variety of encapsulation methods available, they may be used to meet specific goals and types of enzymes and biotechnology processes (Bahrami et al., 2020).

**TABLE 1:** Basic description and applications of solid support free encapsulates.

Encapsulation system	Basic characteristics	Applications	References
Lipid contained particles	Lipid natural material, emulsifiers, and co-emulsifiers, as well as water and bioactive chemicals, make up lipidic nanoparticles. They have an incomplete crystallization that leaves holes in their structure that fat-soluble enzymes can fill	Improves plant growth Food preservation disease defense, and nutraceutical uses are all possible	Hosseini et al. (2021)
			Lammari et al. (2021)
Micelles and Polymeric micelles	Polymeric micelles are nano-sized (less than 100 nm) drug delivery devices with a core-shell structure formed by amphiphilic block copolymers self-assembling in aqueous solution. Fundamental properties of micelles concentration, size, surface charge, and morphology of crucial micellar concentration, size, and surface charge Due to their unique architectures, they may be used as nanoreactors and are good for enzyme trapping	Nanocarriers for the delivery of anticancer drugs that are poorly soluble	Sedaghat Doost et al. (2020), Thanki et al. (2013), Thipparaboina et al. (2015)
		Antibacterial washing agents for animal carcasses and fresh vegetables, as well as agrochemical encapsulation systems that increase plant bioavailability	Quiñones et al. (2018), Yadav et al. (2019)
Emulsions	Emulsions are made up of two phases: aqueous and oily (water with oil or oil with water), which are stabilized by surfactants or emulsifiers. They include emulsifiers, which aid in increasing solubility and facilitating component release. The emulsions are kinetically stable, isotropic, clear, and free of coalescence and flocculation. The size of the emulsion droplets is generally between 50 and 200 nm. There are several techniques for obtaining them	Food preservation	Rostamabadi et al. (2019), Rehman et al. (2020), Bento et al. (2020), Hsu et al. (2019), Dos Santos et al. (2018), Jiménez-Colmenero (2013)
		Antimicrobial control	
		Carriers the supplements the Vitamin D, E lycopene	
Liposomes	Liposomes are spherical vesicles that range in size from 20 nm to micrometers. Lipid bilayers form its structure, with polar groups organized in the interior and exterior aqueous phases. Depending on their nature, enzymes might be confined in membranes or inside vesicles	Carriers for compounds pharmaceutical and cosmetic	Akbarzadeh et al. (2013)
		They are used to safeguard the functioning of antioxidants, antimicrobials, bioactive components, and tastes in the agricultural and food sectors	Lammari et al. (2021)
Bilosomes and Ethosomes	Etosomes are liposomes that contain a lot of ethanol (up to 45 percent)	Encapsulate and improve medication transport through the skin for both hydrophilic and lipophilic medicines	Abdulaziz et al. (2015), Zhou et al. (2018), Nasri et al. (2020), Amarachinta et al. (2021)
	They are soft, pliable vesicular structures composed of amphipathic phospholipids organized in one or more concentric bilayers that enclose several water compartments	As a carrier for the treatment of inflammatory infections of the skin	
	Etosomes differ from other lipid nanocarriers in terms of bilayer fluidity, penetration mechanism, and ease of production	Because of their capacity to withstand enzymes and bile salts in the gastrointestinal system, are utilized in the delivery of oral vaccinations	
	Bilosomes are formed when bile salts are added to the vesicles of liposomes and niosomes		
	Bile salts aid in the stability of the bilosome membrane		
Niosomes	Can be unilamellar, oligolamellar, or multilamellar	Distribution carriers drug oral, topical, transdermal, ophthalmic, intravenous, pulmonary	Seleci et al. (2019)
	They are nonionic surfactants with two-layer structures that are thermodynamically stable. These two-layered structures, have a hollow area in the middle. Enzymes that are both hydrophilic and hydrophobic can be enclose. Entrapment of hydrophilic enzymes in niosomes might take either in the core aqueous domain or on the bilayer’s surface when the enzymes enter the structure		Khoee and Yaghoobian (2017)
			Bhardwaj et al. (2020)
Layersomes and Transfersomes	Layersomes are structures created by layering oppositely charged polyelectrolytes upon regular liposomes, which may enhance storage stability, robustness, and the ability to get high enzyme encapsulation. A phospholipid bilayer plus an edge activator make up transferomes	Insulin, bovine serum albumin, vaccinations, and other proteins and peptides are carried by them. Transferosome formulations are employed for efficient delivery of non-steroidal anti-inflammatory drugs like ibuprofen and diclofenac because to their strong penetration power and flexibility	Buschmann et al. (2021), Sun et al. (2021a), Sun et al. (2021b)
	They are also extremely biocompatible and can transport enzymes of various solubilities		

### Polymeric Micelles

Polymeric micelles, or aggregation colloids generated in solution by the self-assembly of amphiphilic polymers, are a novel method for overcoming a variety of drug delivery difficulties, ranging from low water solubility to poor drug permeability through biological barriers.

Amphiphilic di-block copolymers (polystyrene and poly (ethylene glycol)) and triblock copolymers (poloxamers) are the most widely utilized polymers for micelles production, although graft (e.g. chitosan) and ionic (poly (ethylene glycol)) polymers are also employed (ethylene glycol) The copolymers–poly (-caprolactone)-g-polyethyleneimine) are utilized. The hydrophilic segment is usually made of PEG (polyethylenglycol), but other polymers such as poly (vinyl pyrrolidone), poly (acryloylmorpholine), or poly (trimethylene carbonate) can also be used; the hydrophobic segment can be made of poly (propylene oxide), polyesters like poly (-caprolactone), or glycolic and lactic acid polymers and co-polymers (Mandal et al., 2017). Among all of these options for joining hydrophobic and hydrophilic blocks to generate amphiphilic polymers, attaching phosphatidyl ethanolamine (PE) to the hydrophilic PEG is a comparatively easy technique that only requires one conjugation step between amine or acid terminated PE. The synthesis has good structural control, has a good lipophilic hydrophilic balance, is chemically stable, biocompatible, and biodegradable. Other block co-polymeric assemblages have more structural flexibility and can hold more drugs (Logie et al., 2014).

Depending on the technique of manufacture and the physicochemical features of the medication, protein can be encapsulated in the micelles during their creation or in a subsequent stage. Direct dissolving is the most straightforward way of preparation; additional options include dialysis, emulsion with solvent (or co-solvent) evaporation, and solution-casting followed by film hydration. The choice of approach is based on both polymer and drug features, and further information may be obtained in specific reviews (Makhmalzade and Chavoshy, 2018). Because micelle features such as polarity and hydration degree are not uniform inside the carrier, the medication can be housed in a variety of locations, such as near the surface or deep within the carrier, depending on its qualities. Hydrophobic medicines are usually loaded and stored in the inner core.

Polymeric micelles are attractive carriers for many administration routes due to their small size, ease of synthesis, and good solubilization capabilities. Micelles are thermodynamically self-assembled entities with reversible limits that may be disassembled by a variety of destabilizing forces. Micelles must deal with varied challenges depending on the route of administration; the behavior of the micelles is then determined by their composition and general features, such as size, surface charge, shape, thermodynamic and kinetic stability. All of these properties are crucial in determining the micelle’s destiny and, as a result, drug bioavailability.

Polymeric micelles offer a number of characteristics that make them a viable protein delivery platform. In comparison to lipid-based vesicles, they are nano-sized, monodispersed, reasonably stable, and cost-effective, and they may be surface-modified or stimuli-sensitized by changing their chemical structure. Many amphiphilic polymers have been produced and studied for their capacity to self-assemble and load poorly soluble proteic drugs throughout the years due to their lower size compared to other lipid carriers. The creation of the amphiphilic polymer has been connected to various combinations of hydrophobic and hydrophilic polymeric building units.

Hydrophobic protein might be loaded into the center of polymeric micelles. The physical interaction between the proteins and the polymers, on the other hand, governs the trapping of proteins in the micelles’ core. As a result, a variety of options for conjugating hydrophobic and hydrophilic blocks have evolved in order to develop a better micellar system capable of loading a specific chemotherapeutic drug optimally. The options for conjugating polymeric blocks are limitless, and many different combinations have been tested in recent years.

### Lipid-Based Nanocarriers

Lipid-based nanocarriers are effective drug delivery nanosystems. The remarkable qualities of their lipid components, such as high flexibility, biocompatibility, and low toxicity profile, have drew a lot of attention to these nanocarriers in recent decades. Lipid-based nanocarriers can be used to improve the therapeutic potential of medications with biopharmaceutical constraints, such as limited water solubility or stability, as well as to provide alternatives to the parent route.

Liposomes, self-nano and microemulsifying drug delivery systems, nanoemulsions, and nanocapsules are all examples of lipid-based nanocarriers. They all have in common the ability to alter their formulation to the features and requirements of the oral route. Indeed, the majority of the oils and fats employed in the production of these nanocarriers are derived from dietary lipids, allowing for improved oral permeability and biodegradability. Furthermore, lipid-based nanocarriers may be engineered to interface with particular cell populations in the gastrointestinal system, boosting medication delivery effectiveness (Ghorbanzade et al., 2017; Plaza et al., 2021).

### Liposomes

Liposomes are spherical vesicles made up of one or more lipid bilayers made up of natural or manufactured phospholipids that form lipid bilayer spheres in water and encase aqueous nuclei. Phospholipid molecules and cholesterol with mixed lipid chains (such as phosphatidylcholine from soybeans or phosphatidylethanolamine from eggs) can be found in liposomes (Colletier et al., 2002; Liu et al., 2020). Amphiphilicity of phospholipids is responsible for the production of vesicles in aqueous solutions, which are stabilized by hydrogen bonds, van der Waals forces, and electrostatic interactions (Sercombe et al., 2015; Pattni et al., 2015).

Liposomes come in a variety of sizes, ranging from 20 nm to several micrometers, and are commonly employed as biomembrane models and biocompatible delivery methods (Zarrabi et al., 2020). Liposomes are tiny bilayer vesicles that come in a variety of sizes and forms. They’re lipid layer vesicles that enclose an aqueous phase.

**FIGURE 3:**
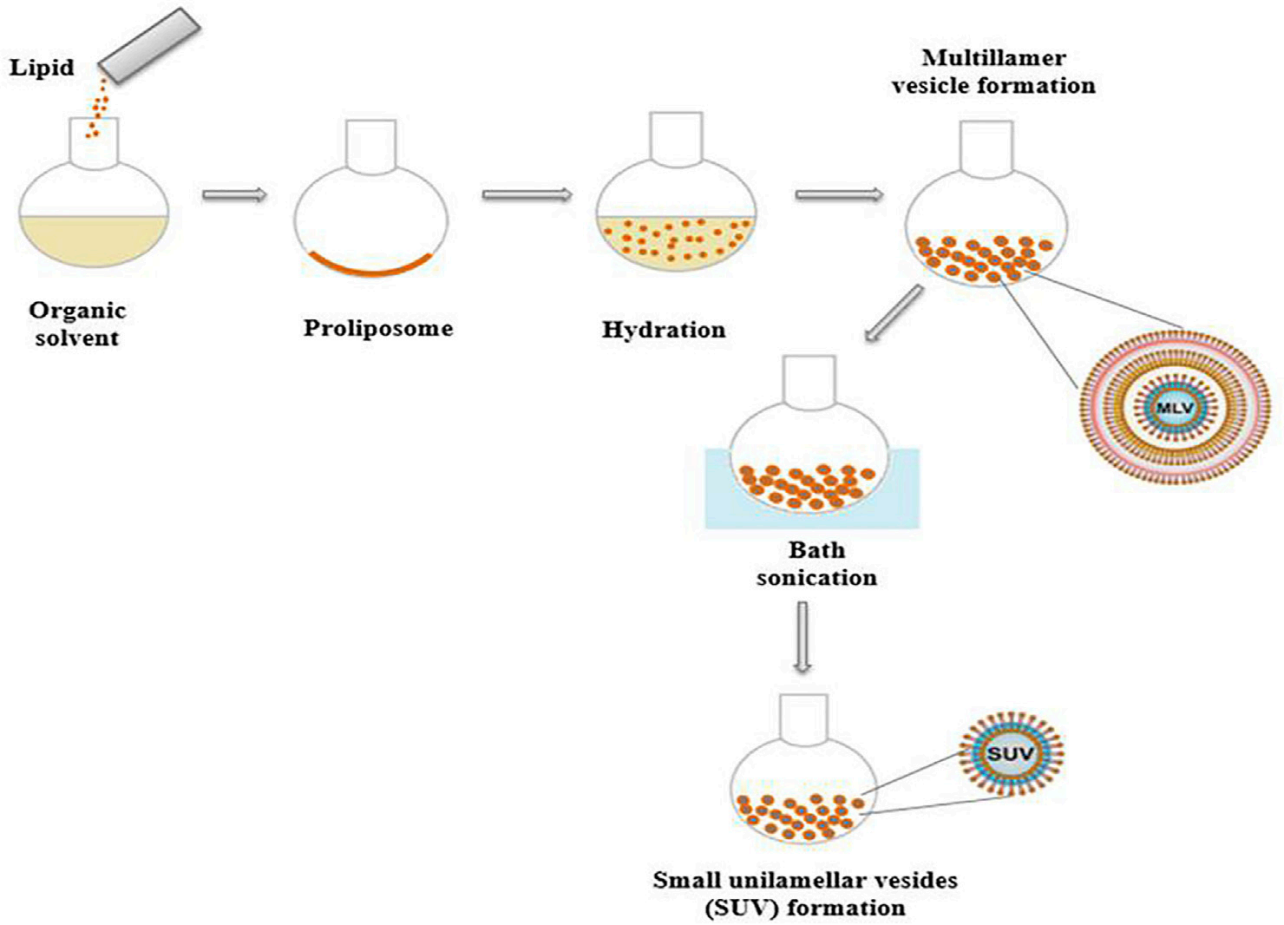
Schematic representation of liposome preparation.

Liposomes may be classified into several kinds based on their fabrication technique, which may include one or multiple bilayer shells (Figure 3). Liposomes are divided into four types based on their structure: multilamellar vesicles (MLVs, 0.1–15 mm), small unilamellar vesicles (SUVs, 15–50 nm), large unilamellar vesicles (LUVs, 100–1 mm), and multivesicular vesicles (MVVs, 1.6–10.5 mm), OLVs are oligolamellar vesicles (OLVs) having a diameter of 100–1,000 nm. A liposome with an aqueous phase enclosed by a bilayer structure is known as a unilamellar liposome. The quantity of aqueous phase contained in each mole of lipid rises as particle size increases, and stability improves (Doskocz et al., 2020; Liu et al., 2020). Static and dynamic light scattering (DLS), microscopy, size exclusion chromatography (SEC), field flow fractionation (FFF), and centrifugal analysis are used to evaluate the size distribution of liposomes. Electron microscopy or spectroscopic technologies can be used to examine the multilayer structure of liposomes (Waglewska et al., 2020).

Because of their advantages such as lipophilic/hydrophilic properties, ability to compartmentalize space, and flexible colloidal sizes, liposomes have been widely used to entrap various compounds such as peptides (Mohan et al., 2016), enzymes (Jahadi et al., 2015), antioxidants (Tavakoli et al., 2018), antimicrobials (Cui et al., 2017), essential fatty acids (Faridi Esfanjani et al., 2018).

The ratio of the substance encapsulated in the liposomes to the total amount of compound at the initial input is known as encapsulation efficiency. It is dependent on the liposome’s capacity to capture the encapsulated molecules as well as the number of encapsulated molecules’ starting moles. Depending on their solubility and polarity, encapsulated compounds interact with liposomes in a variety of ways. They can be completely trapped in the lipid chain’s bilayer area, interspersed in the polar head group region, adsorbed on the membrane surface with a hydrophobic tail, or imprisoned in the interior aqueous compartment (Gomez and Hosseinidoust, 2020). Dilution with alcohol, extraction of lipids in a chloroform solution, or forced bilayer breakdown by surfactants are all common methods for determining the quantity of substances contained in liposomes, with subsequent measurement of release (Tomadoni et al., 2019; Amarachinta et al., 2021).

Quantitative approaches such as spectrophotometry, fluorescence spectrometry, enzymatic methods and electrochemical methods are all dependent on the liposome vesicle’s characteristics (Gerente et al., 2007).

Liposomes are a good way to preserve labile molecules like enzymes from degradation while yet allowing them to function effectively. It’s a very effective approach for physically immobilizing enzymes, which makes them more stable and allows them to be introduced into cell membranes with regulated release of active principles (Mikhaylov et al., 2011).

Enzymes, peptides, and nanoparticles may now be encapsulated within liposomes thanks to advances in entrapment techniques. Encapsulation techniques preserve enzyme function by preserving it from denaturation caused by pH extremes, high temperatures, and protease action (Ngo et al., 2010).

Liposomes’ physical, chemical, and biological stability are only a few of the issues that restrict their use. Dimensional stability, lipid-to-substance ratio, and entrapped membrane are all examples of physical stability. Liposomes’ physical stability can be enhanced by storing them at low temperatures (Babaki et al., 2016). Liposomes are typically stable for minutes to weeks. Using specific procedures, however, liposomes may be bonded to be stable for extended periods of time. Tan et al. (2016), for example, found that unilamellar liposomes produced by the emulsion transfer method can last up to 26 days. The hydrolysis and oxidation of lipids are the primary causes of chemical instability. It’s also brought on by the digestion of catabolic enzymes. Hydrolysis is used to eliminate fatty acid residues. Oxidation also has an impact on unsaturated lipids. The biological stability of liposomes is now a hot issue; for example, it is affected by the type of lipids used to create the liposomes, as well as polymerization and interactions with other components. Optical techniques based on solute leakage from liposomes have been used to evaluate membrane mobility and penetrability using common fluorescent probes (Mendes et al., 2011). Chen et al. (2017) used changes in the Z-average diameter and retention rate to assess the stability of liposomes loaded with b-ionone after storage at 4°C.

Traditional liposomes are commonly used to immobilize enzymes and increase their stability for storage or usage. Encapsulation in liposomes, on the other hand, can reduce the bioavailability of encapsulated enzymes (Nguyen et al., 2016).

As a result, numerous attempts have been undertaken to alter the outer membrane of liposomes in order to improve their stability and permeability, with the goal of developing innovative uses as a result (Kim et al., 2014).

### Ethosomes

The ethosomes are elastomeric nanovesicles made of phospholipids with a high ethanol concentration (20–45%). (Waglewska et al., 2020). Both hydrophilic and hydrophobic components can be encapsulated in these easily produced lipid vesicles. Essential oils may be integrated into ethosomes by dissolving them in ethanol with great flexibility and entrapment efficiency because ethosomes have a shell of bilayer phospholipids and a core of ethanol phase. Because of ethanol evaporation, the fundamental disadvantage of ethosomes is their limited stability. Because of their increased permeability qualities, most of the current literature on the application of essential oils loaded ethosomes is in the field of topical distribution. This is owing to their high fluidity, which is attributable to the synergistic impact of phospholipids and ethanol. Ethanol is a permeation enhancer that has been used to make these elastic nanovesicles in vesicular systems. These ethosomal systems are more effective in releasing chemicals to the skin than liposomes or traditional hydroalcoholic solutions. Ethosomes are efficient in transporting chemicals into the bloodstream. It’s been utilized to deliver the antiviral medication acyclovir via the skin. Because of their superior water solubility and biocompatibility, ethosomal gels are used with medicines. (Ascenso et al., 2015; Yücel et al., 2019).

Ethosomes are non-invasive delivery carriers that allow medications to reach deep into the epidermal layers or the systemic circulation (Kumar et al., 2020). They offer outstanding features such as room-temperature stability, high entrapment efficiency, and compatibility with the stratum corneum, which allows for efficient release of hydrophilic and lipophilic medicines into the deep layers of the skin (Abdulbaqi et al., 2016; Xiao-Qian et al., 2019). According to their composition, ethosomals systems may be divided into three groups:

• Phospholipids, a high concentration of ethanol (45%), and water make up traditional ethosomes.
• Drugs entrapped in typical ethosomes have molecular weights ranging from 130.077 Da to 24 kDa.
• Another type of alcohol was added to create binary ethosomes; the most common alcohols are propylene glycol and isopropyl alcohol.

### Transferosomes

Transfersomes, also known as elastic, deformable, or ultra-flexible liposomes, were first reported by Cevc and Blume (1992). Their characteristics make them excellent transdermal medication delivery devices (TDDS). Due to their ultra-deforming membrane, drugs are easily administered into or through the skin, squeezing themselves along the intracellular lipid or transcellular channel of the stratum corneum. Furthermore, owing of their flexibility, they have a lower risk of skin rupture, and this trait also allows them to follow the natural water gradient across the *epidermis* under no occlusive circumstances (Opatha et al., 2020).

Transfersomes hydrophobic and hydrophilic compositions allow them to transport medications of various solubilities while being extremely biocompatible owing to their inherent phospholipid content (Manconi et al., 2019). They may also contain low or high molecular weight medicines, with an entrapment effectiveness of up to 90% (Abdellatif and Abou-Taleb, 2016). These structures also have the advantages of being resistant to metabolic breakdown and releasing their contents slowly and gradually. However, transfersomes have some drawbacks, such as oxidative deterioration and the challenge of transporting hydrophobic medicines without affecting their elastic and deformability features (Harmita et al., 2020).

### Niosomes

Niosomes are non-ionic surfactant vesicles that are formed by hydrating synthetic non-ionic surfactants with or without cholesterol or lipids (Patra et al., 2018). These are generated by non-ionic surfactant self-assembly in non-aqueous fluids as spherical, multilamellar, and polyhedral structures, as well as inverse forms that only emerge in aqueous solvent. A variety of non-ionic surfactants have been utilized to produce vesicles since then. Polyglycerol alkyl ethers, glucosyldialkyl ethers, crown ethers, polyoxyethylene alkyl ethers, ester linked surfactants and steroid linked surfactants (Zhu et al., 2018).

Niosomes are a handy, long-lasting, targeted, and effective medicine delivery mechanism. They may be used to encapsulate a variety of natural active compounds, such as enzymes and peptides. These vehicles, unlike liposomes, have a unique structure that permits them to transport both hydrophilic and lipophilic medications, making them a viable drug delivery option (Manosroi et al., 2016).

Niosomes are biocompatible, biodegradable, and long-lasting, allowing for controlled and sustained drug administration to the target site (Habib and Singh, 2021). Papain is a proteolytic enzyme that is generated from the latex of the *Carica papaya* plant and is used to repair wounds. Despite this, the enormous molecular weight of papain makes it challenging to employ topically (Ruslan and Roslan, 2016). They are vesicular structures that look like liposomes and can transport both amphiphilic and lipophilic medicines. Vesicles are defined as little sac-like entities that are made up of or related to. Both niosomes and liposomes have the same medication delivery capacity and boost effectiveness when compared to free drug. Niosomes are chosen over liposomes because they are more chemically stable and cost less. In aqueous environments, non-ionic surfactant vesicles are tiny lamellar structures including spherical, uni or multilamellar, and polyhedral vesicles with sizes ranging from 10–1,000 nm.

The mean diameter of niosomal vesicles is calculated using the laser light scattering technique, and their shape is intended to be spherical. Electron microscopy, optical microscopy, ultracentrifugation, molecular sieve chromatography, photon correlation microscopy, and freeze fracture electron microscopy may all be used to quantify diameter. Under light polarisation microscopy, an X–cross formation is used to quantify bilayer formation. Nuclear magnetic spectroscopy and electron microscopy are used to count the number of lamellae.

### Bilosomes

Bilosomes (bile salts including niosomes) are a novel type of vesicular carrier that was initially reported by Conacher et al. (2001). Bilosomes are non-ionic amphiphile closed bilayer vesicles that integrate bile salts, similar to niosomes. Several studies have shown that bilosomes may be used to administer vaccinations successfully orally (Aburahma, 2014; Jain et al., 2014). Bile salts in the lipid bilayers of bilosomes make them more resistant to bile salts and enzymes found in the gastro-intestinal (GI), providing protection for the entrapped vaccine from the GI tract’s hostile environment.

In terms of composition, chemical stability, and storage conditions, they differ from liposomes and niosomes. Vaccines based on bilosomes provide a systemic and mucosal immune response that is comparable to the immunological response elicited by the subcutaneous method. When it comes to storage and handling, bilosomes don’t require any specific considerations. Bilosomes have the advantage of allowing little amounts of antigen to be effective while also increasing the efficacy of antigen that is weak initially injected. They are a safe and effective alternative to conventional vaccinations since they do not involve the use of live organisms. This non-invasive technology has a higher acceptability and compliance rate among users.

Vesicular delivery systems are highly organized assemblies made up of one or more concentric bilayers that develop because of amphiphilic building blocks self-assembling in water. These systems are crucial for targeted medication delivery because they have the capacity to localize drug activity to the organ or site of action, lowering concentrations at other parts of the body, the drug delivery systems (DDS) has the ability to maintain drug levels at a preset pace (zero-order kinetics) and maintain effective medication concentration inside the body, therefore reducing adverse effects.

Encapsulation technologies are used to produce controlled-release delivery systems in medicine, pharmaceutics, agriculture, and cosmetics. Drugs, insecticides, perfumes, and enzymes are released over time via colloidal systems including ethosomes, bilosomes, and niosomes. The composition of colloidal particles used to encapsulate enzymes and other proteins determines the properties of the particles. The active principle’s capacity to encapsulate, protect, and distribute the active principle is influenced by the composition of encapsulated systems (Perry and McClements, 2020).

Colloidal vesicles may be made from a variety of edible components, including proteins, polysaccharides, lipids, phospholipids, and surfactants (Aditya et al., 2017). As a result, choosing the best components to build an enclosed system is critical.

For specific applications, the size and shape of the colloidal enzyme-encapsulating vesicles can also be modified. Depending on the components utilized and the manufacturing techniques used to produce them, colloidal particles can range in size from around 10 nm to 1 mm. Colloidal particles are typically spherical, although various morphologies, such as ellipsoid, cubic, fibrous, or irregular, are also conceivable. Emulsions are mixtures of oil (O) and water (W). It is possible to make O/W and W/O emulsions, both of which are appropriate for encapsulation (Niu et al., 2017). As a result, O/W emulsions are used to encapsulate enzymes in oil-filled capsules for oral delivery. This sort of technology is used to preserve bioactive peptides, enzymes, and proteins from digestive enzyme breakdown. The fact that these systems may be made from a variety of natural oils and emulsifiers is their major benefit. W/O emulsions, on the other hand, are more expensive and time-consuming to make. Furthermore, they are unstable, decomposing during storage or when subjected to environmental stressors (Sobczak et al., 2017; Perry and McClements, 2020).

## Liposome Application for Laminarinase and Chitinase Encapsulation

Chitinases (EC 3.2.2.14) are hydrolytic enzymes that can dissolve the 1, 4-glycoside bonds in chitin, which is a major structural component of insect exoskeletons and fungal cell walls. Chitinases are a diverse collection of enzymes that differ in structure, substrate selectivity, and method of action. These enzymes have a size range of 20 kDa to roughly 90 kDa. Chitin is the second most common biopolymer on the planet, consisting of a linear polymer of -1, 4-N-acetylglucosamine (GlcNAC).

The hydrolysis of 1,3- and 1, 4-linkages in -D-glucans is catalyzed by laminarinase (EC 3.2.1.6, also known as 1,3-glucanohydrolase or -1,3-glucanase). Laminarinase principal hydrolytic activity is on the 1, three- glucose polymer laminarin. Laminarin is a low molecular weight -glucan storage polysaccharide found in fytopategens’ cell walls.

Laminarinases and chitinases are important enzymes responsible for the lysis of the fungal cell and sclerotial wall in fungi such as *Trichoderma spp* (Koteshwara et al., 2021). Different phospholipids, such as soya lecithin, which is made up of various triglycerides, phospholipids (phosphatidylinositol, phosphatidyl choline, phosphatidyl ethanol amine), and glycolipids, can be utilized to make liposomes (Jørgensen et al., 2004; Li et al., 2015).

The thermodynamic characteristics of the encapsulation process of both enzymes in soybean licithin liposomes were compared by Cano-Salazar et al. (2011). Liposomes were made using the Bangham method (Bangham, 1993): soybean licithin was dissolved in chloroform; then organic solvent was evaporated, obtaining thin lipid layer in flask surface; lipids film hydration was carried out with enzyme-contained aqueous solution by hand shaking.

According to Ávila et al. (2003), this method leads to obtaining MLV that are confirmed by microscopy. The molal partition coefficients (Ko/w) were determined using the technique (Lozano and Martínez, 2006). The standard free energy of transfer (Gwo) from aqueous medium to organic system was estimated using the technique of Ávila et al. (2003). To acquire data on the enthalpy of transfer (ΔHw→o), the temperature dependency of partitioning (van’t Hoff technique) was used. The entropy of transfer (ΔSw→o) was calculated using van’t Hoff linearization and the equation ΔSw→o = (ΔH w→o - ΔG w→o)/T.

The term “partition” refers to the distribution of an enzyme between two phases in a dynamic equilibrium. Because the solute is spread between two different phases: water (w) and liposomes (o), it is a heterogeneous equilibrium. The reduction of the enzyme’s concentration in the aqueous phase following liposome formation, which is connected to the partition process between these two phases, may be regarded evidence supporting the distribution process of the enzyme (Dobreva et al., 2020).

**FIGURE 4:**
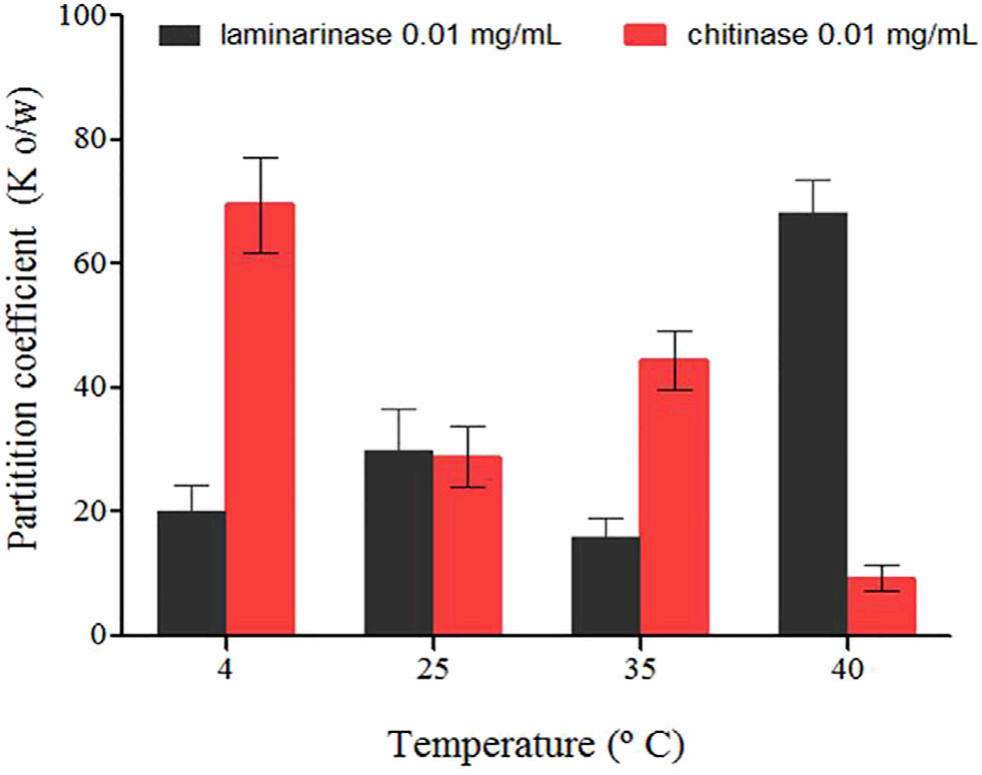
Partition coefficients of enzymes (laminarinase and chitinase) in soybean lecithin liposomes system quantified for different temperatures (±0.1°C), in molality (±standard deviation).

The temperature dependency of the partition coefficients for laminarinase and chitinase in the investigated systems is depicted in Figure 4. In chitinase-contained systems, the Ko/w values decreased with increasing temperature, but for laminarinase microencapsulation, they rose (Figure 4). The partition coefficients (Ko/w) of the enzymes laminarinase and chitinase were larger than one, suggesting that the enzymes have an affinity for microencapsulation in liposomes. However, the thermodynamic characteristics of the microencapsulation of each enzyme vary, which could be due to differences in their primary structure and the amount of amino acids of a lipophilic nature (Nobe et al., 2004).

Table 2 summarizes the thermodynamic functions involved in the transfer of laminarinase and chitinase from aqueous media to soybean lecithin liposomes. ΔG w→o values at 25°C are comparable and negative in both instances. This shows that each enzyme prefers the organic phase, indicating that enzyme transfer from an aqueous medium to an organic environment is not inhibited (Ávila et al., 2003).

**TABLE 2:** Thermodinamic parameters (free energy, enthalpy and entropy) for the transfer of enzymes (laminarinase and chitinase) from aqueous media to soybean lecithin liposomes (Cano-Salazar et al., 2011).

Enzyme, mg/mL	Laminarinase at 0.01 mg/ml	Chitinase at 0.01 mg/ml
ΔG_w→o_, kJ/mol	−8.4	−8.3
Δ H_w→o_, kJ/mol	19.4	−39.3
ΔS _w→o_, J/(mol x K) from equation	93.4	−103.9

Enthalpic and entropic changes refer to energy needs and molecular randomness (increase or decrease in molecular disorder), respectively, resulting in the net transfer of enzyme from the water to the organic phase (Lozano and Martinez, 2006). The sign of the ΔS w→o values defined for chitinase immobilization and laminarinase microencapsulation in soybean lecithin liposomes is different: positive for chitinase immobilization and negative for laminarinase microencapsulation (Table 2). Chitinase transfer enthalpy (ΔH w→o) is negative, while laminarinase transfer enthalpy is positive. As a result, the process is both exothermic and endothermic. The presence of a strong interaction between chitinase molecules and soybean lecithin phospholipids is indicated by a negative enthalpy. Phospholipids can form hydrogen bonds as a hydrogen donor or acceptor (Ávila et al., 2003). The initial cavities filled by the protein in the aqueous phase are now occupied by water molecules once a specific number of enzyme molecules have migrated from the aqueous to the liposome organic phase.

Due to water-water interactions, this occurrence is accompanied by the release of energy. However, depending on the molecular structure of the enzyme, it’s also important to remember that water molecules can form a ring around the enzyme’s hydrophobic aminoacids (hydrophobic hydration). This occurrence is followed by an energy intake as well as an increase in local entropy due to the separation of certain water molecules (Lozano and Martinez, 2006).

Table 2 reveals that transfer mechanisms from water to lecithin liposomes were endothermic for the laminarinase, implying large increases in the system net entropy. Only the laminarinase-containing system has positive transfer entropies (S w→o). The disorder created in the hydrophobic core of the lipid layers during the separation of the phospholipids hydrophobic tails to accommodate the protein molecules in liposomes may account for the rise in entropy during the transfer of laminarinase to lecithin liposomes. The obtained findings show that laminarinase transmission is entropy driven owing to a positive entropy value, whereas chitinase transfer is enthalpy driven due to a negative entropy value (Ávila et al., 2003).

Thus, laminarinase and chitinase microencapsulation occur via thermodynamically distinct mechanisms that should be considered when optimizing and designing encapsulation processes. Ávila et al. (2003) used optical light microscopy (40X) to count the liposomes immediately after production and every 10th day throughout storage at 4, 25, and 40°C.

**FIGURE 5:**
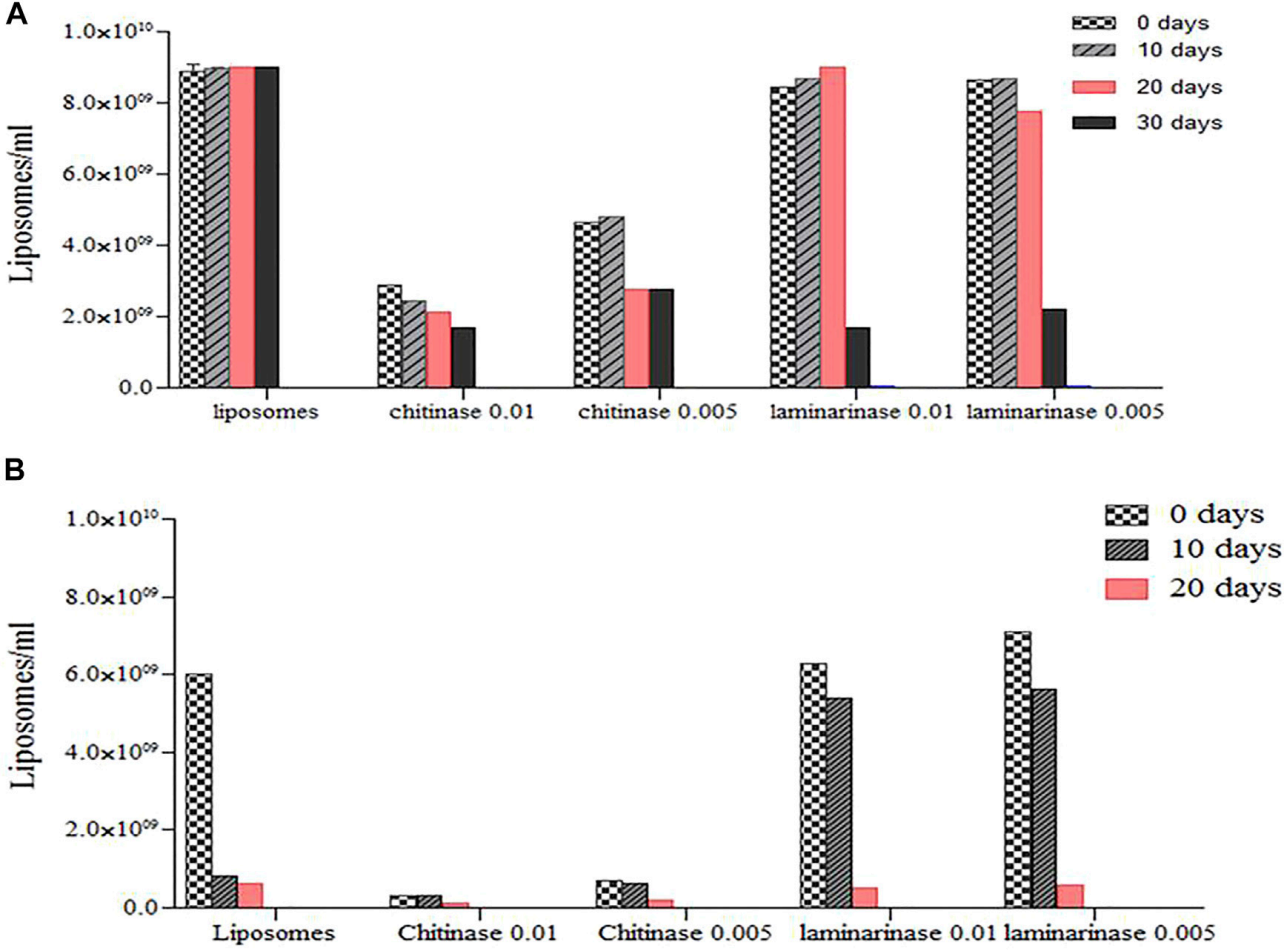
Concentration of enzyme (Chitinase at 0.01 mg/ml and Laminarinase at 0.01 mg/ml) loaded liposomes during long-term (10, 20 and 30 d) storage at diferents temperature: **(A)**, T−4°C and (B), T−25°C. Quantification was performed by optical light microscopy (40X) (Cano-Salazar et al., 2011; Pérez Molina et al., 2011).

The presence of enzymes and rising temperatures during liposome synthesis resulted in a reduction in their quantity and storage stability over time (Figure 5). Significant decreases were detected in the 20th day in the presence of chitinase at both storage temperatures, with disappearance at 40 and 30 days for 4°C and 25°C, respectively. The number of chitinase-containing liposomes formed at 25°C (Figure 5) was substantially smaller than the number of liposomes obtained without enzymes or in the presence of laminarinase, which appears to be related to distinct interaction processes (Cano-Salazar et al., 2011).

At 4°C, the concentration of laminarinase was equal to that measured in the system without enzymes, and it was higher than found at 25°C. For 4 and 25°C, significant decreases were seen in the 10th and 20th days, with disappearance after 50 and 30 days, respectively. Liposome numbers did not fall as much at 25°C in this case as they did in liposomes without enzymes. The interaction of proteins and lipids, which in the instance of chitinase destabilized the liposomes, whereas laminarinase stabilized them for short periods of contact before destabilizing them, might explain the effects of enzymes on liposome stability.

Liposome systems’ stability is influenced by two factors: 1) the liposome component may degrade due to hydrolysis and oxidation; chemical changes in the layer-forming molecules may affect physical stability; for example, if phospholipids lose one of their acyl chains (turn into their lysoforms), the liposome structure is affected; and 2) changes within the lipid-layer, aggregation, or fusion may affect the physical structure of the liposomes. The use of pure phospholipids can improve storage stability (Bangham 1993; Ávila et al., 2003). As a result, soybean lecithin liposomes are sensitive to the presence of enzymes and should be kept at a low temperature.

The stability of the free and encapsulated enzymes was also tested during storage at 4°C. (Figure 5). The nitrophenyl group of *p*-nitrophenyl-D-N-acetyl-glucosamide served as substrate was colorimetrically measured to evaluate chitinase activity, as stated earlier by Dzakiyya et al. (2020).

The activity of laminarinase was determined using laminarine from *Laminaria digitata* as a substrate, according to Singh et al. (1999). The activity of laminarinase was measured spectrophotometrically using the Somogyi-Nelson technique, which involved monitoring the release of reducing sugar (Nelson, 1944).

**FIGURE 6:**
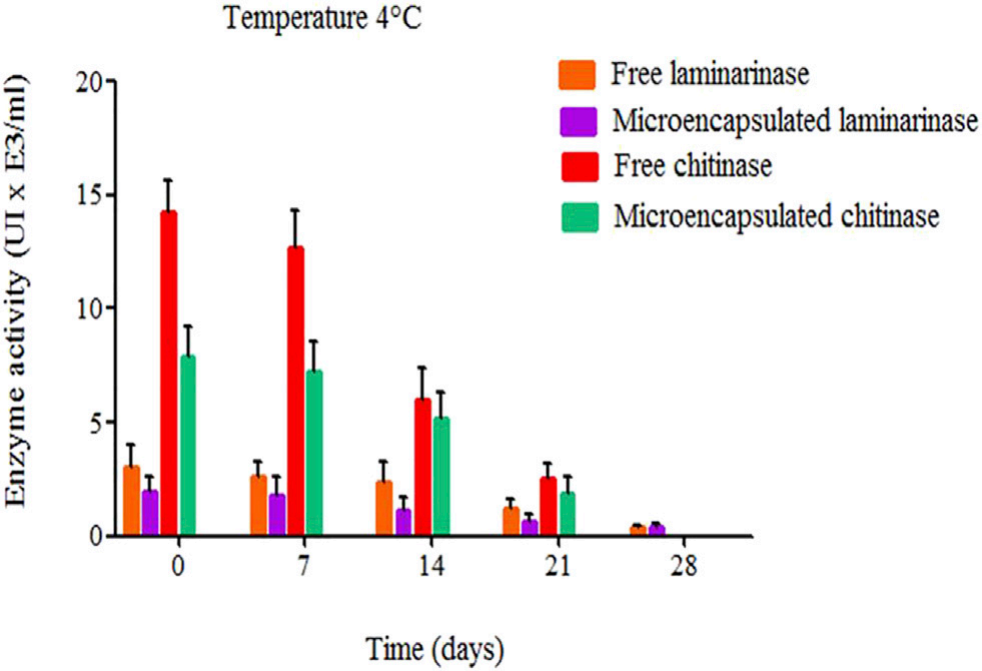
Microencapsulated and free chitinase and laminarinase activity (both at 0.01 mg/ml) during storage at 4°C (Cano-Salazar et al., 2011; Pérez-Molina et al., 2011).

The results in Figure 6 depicts the activity of free and encapsulated chitinase and laminarinase following storage at 4°C. Due to the encapsulation effect, microencapsulated enzymes were less active than unbound enzymes. Chitinase activity was twice as low in the presence of liposomes as it was in the absence of liposomes, whereas laminarinase activity was considerably reduced after encapsulation. Enzyme encapsulation is partially to blame for the decreased activity. In microencapsulated form, the enzyme concentration is lower than in free form (Cano-Salazar et al., 2011; Pérez-Molina et al., 2011).

The enzymes have run into a new issue after being encapsulated, according to Chaize et al. (2009). The lipid membrane’s permeability barrier significantly reduces the activity of the enzyme trapped in the liposome by lowering the rate at which substrate molecules enter the liposome and therefore lowering the substrate concentration inside the liposome. It is possible that this is why chitinase and laminarinase activity drops following microencapsulation.

Frank et al. (2018) found similar results when they tested the enzymatic activity of glucuronidase-loaded liposomes before and after 30 days of storage at 4°C and -80 °C. They found a reduction in activity. Free enzymes lost activity faster than microencapsulated enzymes (Figure 6). Immobilization of laminarinase and chitinase increased the stability of the enzymes (Cano-Salazar et al., 2011; Pérez-Molina et al., 2011).

Finally, it was demonstrated that mycolytic enzymes trapped in liposomes may be used to suppress certain plant infections (Pérez-Molina et al., 2011; Ilyina et al., 2013). This discovery might give an alternative to synthetic chemical fungicides in the future. In the presence of a combination of encapsulated enzymes and enzymes with thiabendazole, a synergistic impact on *F. oxysporum* vitality was shown.

Cano-Salazar et al. (2011) and Pérez-Molina et al. (2011) developed techniques for encapsulating both mycolytic enzymes in soybean liposomes. In the absence and presence of thiabendazole, the enzymatic and antifungal activity of microencapsulated and free enzymes (chitinase and laminarinase from *Trichoderma sp*.) in soil against *F. oxysporum* was assessed. A test against the pathogen *F. oxysporum* was conducted on tomato plants (*L. esculentum Mill*) (Ilyina et al., 2013). Chitinase and laminarinase encapsulated in soybean lecithin liposomes retained an enzymatic activity that allowed *F. oxysporum* to be controlled.

Both enzymes, when used alone and in combination, inhibited fungal growth. In the presence of thiabendazole, microencapsulation improved the enzyme’s stability. The use of these enzymes suppressed fungal growth largely (90–98%) or completely (100%) and reduced the amount of chemical fungicide used chemical fungicide (Ilyina et al., 2013).

The presence of encapsulated enzymes increased the development of tomato plants. As a result, the initial notion of using a mycolytic enzyme immobilized in soybean lecithin liposomes to control infections was verified. This discovery might pave the way for less reliance on synthetic chemical fungicides.

Liposomes have a high value in science because they may be used as a research model to replicate more complicated natural systems since they are regarded a cell membrane-like system for studying protein functioning, including tolerance (Mikhaylov et al., 2011).

Liposomes have been used as a model in studies of pollen, poisons, and pesticide tolerance, as well as transmembrane metabolism in plant organelles. Liposomes are employed as carriers for slow or delayed release pesticide formulations, which help the active ingredient absorb into the plant’s vascular system and are safer for the environment. In veterinary medicine, liposomes are utilized to extend the bioactivity of vaccinations and medicines. Liposomes are the most widely used and well-studied drug carriers, offering considerable therapeutic benefits for the transport of enzymes, antimicrobials, anticancer, and anti-inflammatory medicines (Chaize et al., 2009; Mikhaylov et al., 2011).

## Future Trends on Liposome-Entrapped Enzymes

Enzymes encapsulation in liposomes is a typical method for protecting enzymes from environmental impacts and delivering them to specific locations in the pharmaceutical and food industries. Encapsulated enzymes have grown increasingly popular in recent years. Entrapment technique depends on the influence of enzyme type on the enzyme’s kinetic parameters and modifies its optimal pH and temperature. As a result, the enzyme may be employed in a wider temperature and pH range. Liposomes are an appealing option for enzyme immobilization because of these features.

Liposomes, which are nano-sized phospholipid bubbles, have gotten a lot of interest as drug carriers. Liposomes are simple to make, biocompatible, and can hold a wide range of medicines, DNA, and diagnostic agents. Their *in vivo* qualities are straightforward to manipulate. Many liposomal medications are actively being developed, and some have already received clinical approval. By adding particular ligands to the surface of liposomes, they may be directed to certain tissues. Antibodies and their fragments, folate, transferrin, and specific peptides are being employed as liposome ligands. Liposomes are also effective immunological adjuvants for protein and peptide antigens and are widely used in experimental immunology and for vaccine preparation.

Liposomes’ capacity to solubilize chemicals with difficult solubility qualities, sequester compounds from potentially dangerous environments, and release integrated molecules in a consistent and predictable manner can also be employed in the food processing business. Lecithin and other polar lipids, for example, are commonly isolated from foods like egg yolks.

## Conclusion

Encapsulation in colloidal and vesicular carriers allows the use of enzymes for different purposes, such as mycolytic enzymes used to control phytopathogenic fungi. These different solid carrier free systems represent the new methods to deliver treatments such as enzymes, drugs, toxins and antimicrobials, which have strong links with their applications in medicine, agriculture and livestock. They are becoming hot topics in the development of new treatments. Chitinase and laminarinase have affinity to soybean lecithin liposomes. The findings on the thermodynamic properties of enzymes microencapsulation on liposomes can be considered for process optimization in future studies and applications. Stability of enzyme preparations was increased, as well as their antifungal properties.
